# A 51‐year‐old female presenting with headache and vision loss

**DOI:** 10.1111/head.70130

**Published:** 2026-06-22

**Authors:** Laurel Ovrom, Zachary Pardieck, Randy Christopher Bowen, Neena R. Cherayil

**Affiliations:** ^1^ Department of Neurology Northwestern University Chicago Illinois USA; ^2^ Department of Ophthalmology Northwestern University Chicago Illinois USA

**Keywords:** headache, retina, vasculitis, vision loss

**Abbreviations**
GCA: giant cell arteritis
ION: ischemic optic neuropathy
MRI: magnetic resonance imaging

## PATIENT PRESENTATION

Written informed consent for publication of deidentified information was obtained from each patient and will be retained by the corresponding author.

A 51‐year‐old female with a history of menstrual migraine and endometriosis status posthysterectomy and salpingo‐oophorectomy on estrogen replacement therapy presented to the headache clinic for 3 weeks of a new, constant, progressively worsening bifrontal headache followed by a week of persistent right eye vision loss. She described the headache as a dull ache, radiating from her forehead to her temporal regions bilaterally. This headache was longer and more severe than her prior headaches, which were associated with her menstrual cycle and had never been associated with visual or other aura. She denied worsening with position changes or Valsalva, pulsatile tinnitus, or transient visual obscurations. While closing one eye to apply eye makeup 3 weeks into headache symptoms, she noted a dark scotoma with an irregular margin in the inferonasal quadrant of her right eye without eye/periorbital pain or pain on eye movements. She denied dyschromatopsia, tearing, injection, or change in appearance of the eye. She denied scintillation, fortification spectra, or other positive visual phenomena. There was no decrease or spread of the right eye scotoma over the following days. Left eye vision was unaffected. She denied any jaw or tongue claudication or morning limb girdle myalgia/stiffness. On her initial examination, she had tenderness to palpation over the temples and scalp. Cranial nerve examination was notable for an inferonasal quadrant defect in the right eye and full fields to confrontation in the left eye. Visual acuity was 20/70 in the right eye and 20/20 in the left eye. Color vision was full, and there was no relative afferent pupillary defect. An undilated funduscopic examination revealed normal, well‐perfused optic nerves with crisp margins. The remainder of the neurologic examination was entirely normal.

The patient provided written informed consent for the case and associated images to be published.

## LOCALIZATION AND DIFFERENTIAL DIAGNOSIS

This patient's right eye monocular visual field defect has the most localizing value. Monocular vision loss typically localizes anterior to the optic chiasm, as chiasmal and retro‐chiasmal lesions generally cause deficits in both eyes. Moving anteriorly to posteriorly, possible localizations for monocular inferonasal quadrantanopia include the superotemporal retina and anywhere along the optic nerve extending from the optic nerve head to the optic chiasm.^1^ Notably, one exception to the rule of retrochiasmal lesions causing contralateral homonymous visual field loss is temporal crescent syndrome, which results from a lesion in the anterior calcarine cortex where the nasal and temporal fibers are unpaired causing monocular loss of a 30º–40º crescent of the contralateral peripheral temporal field. This would not apply to our patient, as her visual field loss was inferonasal.^2^

Characteristically, chorioretinal pathologies are painless and associated with photopsia (flashing lights), new floaters, or metamorphopsia (the perception of wavy, warped images) with normal color vision. In contrast, unilateral optic nerve pathologies typically result in loss of color vision, a relative afferent pupillary defect, and can often be associated with eye pain.^1^

Possible chorioretinal pathologies include retinal detachment or tear, focal retinal artery ischemia or retinal vein occlusion, serous macular edema, infectious or autoimmune retinopathies, and choroidal tumors. Optic nerve pathologies include optic neuritis, anterior or posterior arteritic and nonarteritic ischemic optic neuropathy (ION), and compressive or infiltrative lesions of the optic nerve.^1^

Another diagnosis to consider for a patient with headache and vision changes is migraine with aura. In the International Classification of Headache Disorders criteria, migraine with aura is defined as “recurrent attacks, lasting minutes, of unilateral fully‐reversible visual, sensory or other central nervous system symptoms that usually develop gradually and are usually followed by headache and associated migraine symptoms.”^3^ To meet criteria for diagnosis, the patient must have had at least two attacks of fully reversible aura symptoms, which can be visual, sensory, speech and language, motor, brainstem, or retinal. They must also have at least three of the following characteristics: spread gradually over ≥5 min, two or more aura symptoms occurring in succession, individual aura lasting 5–60 min, at least one aura symptom unilateral and positive, and aura accompanied by a headache within 60 min.^3^ Our patient does not meet criteria for this diagnosis for several reasons. First, her visual symptoms were persistent and not fully reversible. They also did not spread gradually over time, she did not have any other aura symptoms, and she did not have any positive symptoms. Therefore, she would not meet criteria for migraine with aura.

Given new vision loss and new headache with scalp tenderness in a patient over the age of 50 years, giant cell arteritis (GCA) rose sharply on the list of diagnoses to consider. GCA can cause vision loss through inflammatory thrombosis of branches of the ophthalmic artery, most commonly the posterior ciliary arteries causing arteritic ION.^4^ Posterior ION, retinal artery occlusion, and posterior circulation strokes are also seen with GCA.^5^

The 2022 American College of Rheumatology classification criteria outlines several requirements that must be met for diagnosis of GCA. Although the criteria are reviewed in this section, it is important to note that the American College of Rheumatology stipulates that alternative diagnoses mimicking vasculitis must be ruled out before applying the criteria. In our patient, alternative diagnoses have not yet been excluded given the lack of dilated ophthalmologic examination, labs, or imaging.^6^

Patient age >50 years old is an absolute requirement for the diagnosis of GCA. Additional clinical criteria for diagnosis include morning stiffness in the shoulders/neck, jaw or tongue claudication, new temporal headache, scalp tenderness, and abnormal examination of the temporal artery (tenderness, diminished pulse, or palpable cord), each of which receives a score of two points. Sudden visual loss receives a score of three points.^6^ Our patient receives seven points in this category for new temporal headache, scalp tenderness, and sudden vision loss. Laboratory, imaging, and biopsy criteria include maximum erythrocyte sedimentation rate ≥50 mm/h or maximum C‐reactive protein ≥ 10 mg/L, which receives three points, positive temporal artery biopsy or temporal artery ultrasound, which receives five points, bilateral axillary involvement, which receives two points, and positron emission tomography activity throughout the aorta, which receives two points.^6^ For classification of GCA, a patient must receive at least six points, which our patient meets. Other diagnoses to consider for new headache and monocular vision loss include elevated intracranial pressure resulting from space‐occupying monocular lesions, infectious or inflammatory meningoencephalitis, and cerebral venous sinus thrombosis especially in context of hypercoagulability with estrogen use. Idiopathic intracranial hypertension is a cause of headache and vision loss but typically manifests with papilledema in younger women of childbearing age.

## ADDITIONAL WORKUP AND TREATMENT

The patient was directed to the emergency department. In the emergency department, she was afebrile with normal vital signs. Erythrocyte sedimentation rate and C‐reactive protein were obtained and were 58 mm/h (age‐adjusted upper limit of normal 30.5) and 21.5 mg/L (upper limit of normal 10), respectively. Laboratory workup was otherwise unremarkable, including complete blood count without leukocytosis or thrombocytosis. Computerized tomography brain was unremarkable. Given the elevated inflammatory markers, temporal artery ultrasound was obtained, which showed normal temporal arteries without a halo sign.

**FIGURE 1:**
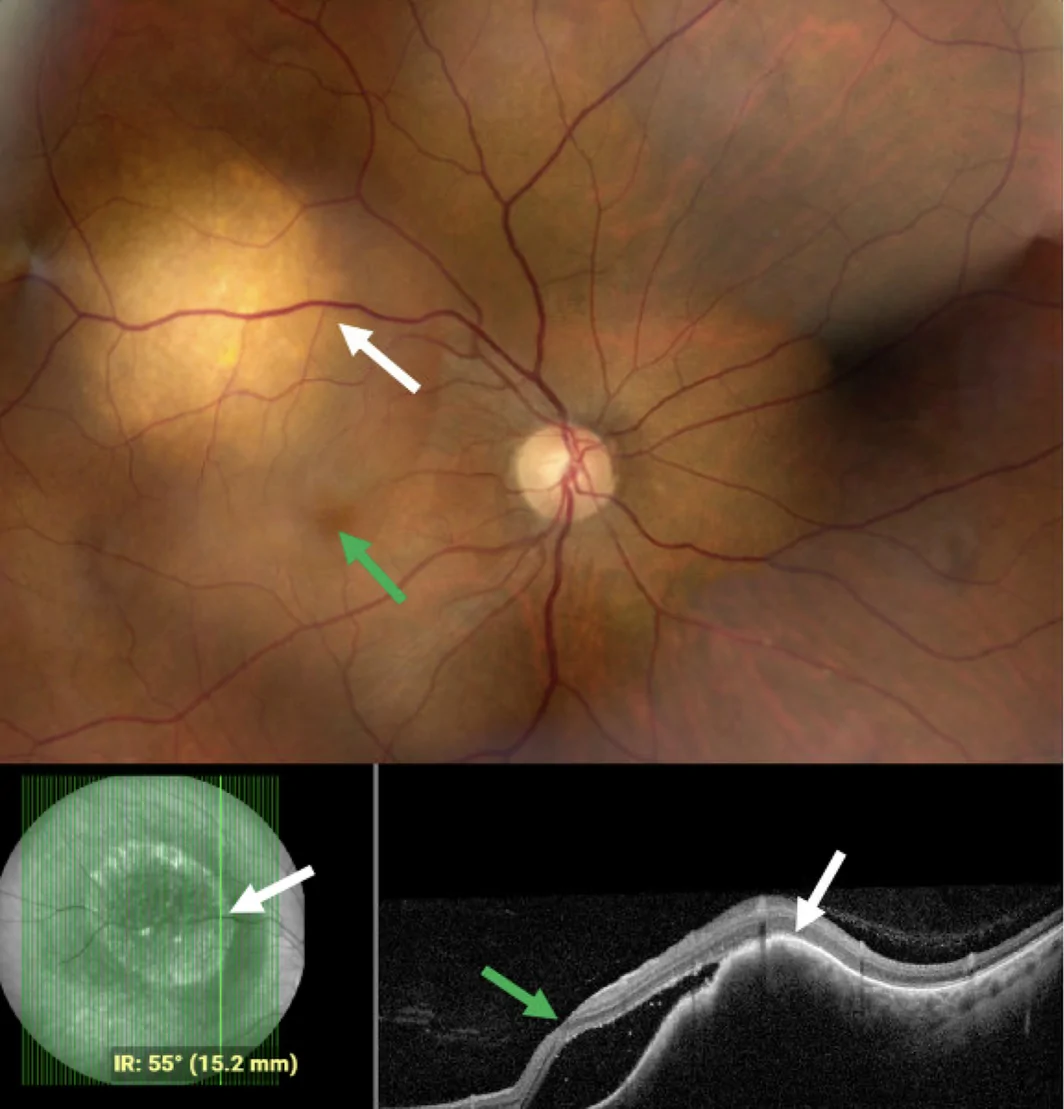
Top: color fundus photograph of the right eye demonstrates an amelanotic creamy‐yellow choroidal mass (white arrow) with surrounding exudative retinal detachment extending through the temporal macula and involving the fovea (green arrow). Bottom: on the left is an infrared (IR) optical coherence tomography (OCT) image with vertical cross‐sectional lines through the retina and choroidal mass. On the right is a 55º OCT image cross‐section corresponding to the IR vertical green line showing an infiltrated choroidal lesion with the apex highlighted by the white arrow with adjacent subretinal fluid causing detachment of the fovea (green arrow). [Color figure can be viewed at wileyonlinelibrary.com]

The patient then underwent a dilated ophthalmologic examination. Notable findings included a 6.5 × 5.5 mm round, elevated, yellow choroidal mass with surrounding exudative retinal detachment in the superotemporal macula (Figure 1). No evidence of macular or extra‐macular ischemia was present, and the remainder of the choroid appeared normal. The patient's inferonasal scotoma was well localized to the area of the choroidal mass and less likely attributable to GCA. A normal temporal artery ultrasound does not fully exclude GCA, and rheumatology initially recommended 1 mg/kg oral prednisone given unexplained elevated inflammatory markers and scalp tenderness. However, given central serous chorioretinopathies can be worsened by steroids, high‐dose IV steroids were deferred.^7^

**FIGURE 2:**
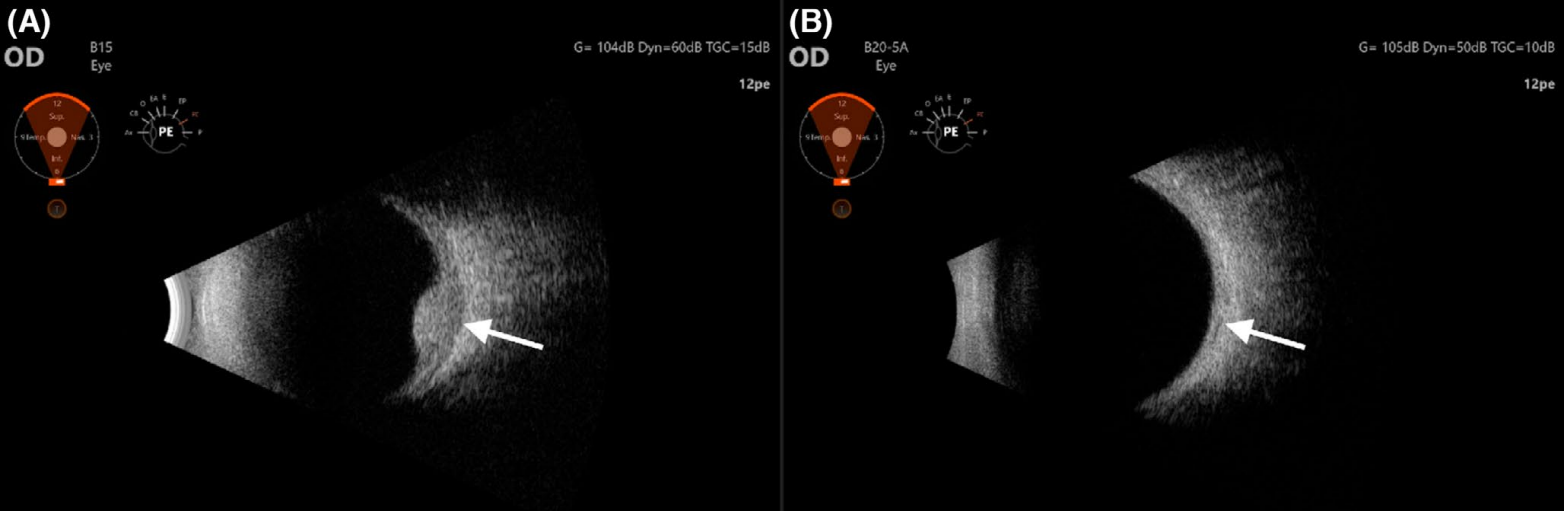
Right eye ocular ultrasound B‐scan. (A) Dome‐shaped choroidal elevated lesion (white arrow). (B) Repeat B‐scan 1 month post initiation of osimertinib shows resolution of previously noted choroidal mass. [Color figure can be viewed at wileyonlinelibrary.com]

**FIGURE 3:**
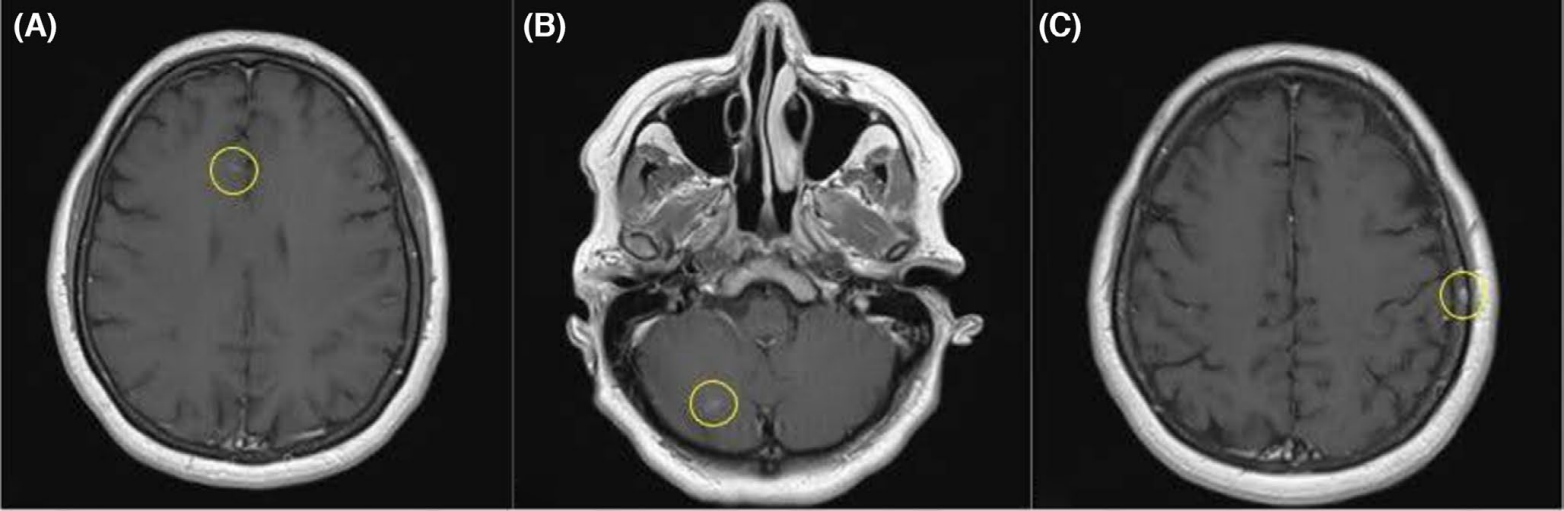
Post‐contrast axial T1 MRI of the brain with yellow circles indicating (A) 0.3 cm focus of enhancement in the paramedian right frontal lobe; (B) 0.4 cm focus of enhancement in the right cerebellar hemisphere; and (C) 1.3 cm left parietal calvarial enhancing and diffusion restricting lesion. None of the lesions are associated with additional signal on GRE/SWI or T2. [Color figure can be viewed at wileyonlinelibrary.com]

A right eye ocular ultrasound revealed a solid mass with focal vascularity, consistent with a malignant choroidal neoplasm (Figure 2A). Primary ocular melanoma was deemed unlikely given lack of pigment and lumpy‐bumpy choroidal appearance on examination, with high suspicion for secondary metastasis. Magnetic resonance imaging (MRI) of the brain and orbits showed several small enhancing parenchymal lesions in the right medial frontal lobe (Figure 3A), temporo‐occipital lobes, and the right cerebellar hemisphere (Figure 3B). There were additionally multiple calvarial lesions concerning for osseous metastatic disease (Figure 3C). These lesions were the presumed etiology for her headaches and scalp tenderness. MRI venogram showed no venous sinus thrombosis. Full body (computerized tomography) imaging showed a 1.9 cm spiculated perihilar left upper lobe nodule, suspicious for a primary lung malignancy, along with an enlarged hilar lymph node and hepatic lesion suspicious for metastases. Biopsy of the spiculated lung mass via bronchoscopy revealed adenocarcinoma with an epidermal growth factor receptor allele variant. She established care with oncology and started osimertinib and bevacizumab for treatment of widely metastatic lung cancer. Follow‐up ocular ultrasound of the right eye 1 month after initiation of therapy revealed resolution of the choroidal lesion (Figure 2B).

## FINAL DIAGNOSIS: ADENOCARCINOMA OF THE LUNG WITH CHOROIDAL, INTRACRANIAL, AND CALVARIAL METASTASES

The choroid is the most common ophthalmic structure affected by systemic metastases, as result of its rich vascularity, which allows for hematogenous spread of disease.^8^ Careful ophthalmic examination and ultrasonography help distinguish choroidal metastases from other causes of choroidal masses (including nevi, hemangiomas, and primary uveal melanomas) based on their shape, color, size, location, and vascularity.^9^ Choroidal metastases are usually creamy white or yellow, dome‐ or plateau‐shaped, and can be unilateral or bilateral.^9^ In an analysis of 1111 patients with metastatic uveal tumors, the primary tumor site was known before detecting the uveal metastasis in 67% of patients.^10^ The most common locations of the primary tumor were the breast (37% of patients) and the lung (26% of patients).^10^ In an analysis of 194 patients with uveal metastases from the lung, the most common symptoms at presentation included blurred vision, pain, and floaters, followed by visual field defects.^11^

The mean life expectancy after detection of uveal metastasis is 12 months.^11^ The most common treatment approaches for choroidal metastases include external beam radiotherapy (30%), observation (22%), systemic chemotherapy alone (17%), and combination of systemic chemotherapy and radiotherapy (15%).^10^ Final visual acuity stabilized or improved in 59% of patients.

## LEARNING POINTS

1. Headaches with vision loss have a broad differential diagnosis that includes primary headache syndromes and many secondary causes including GCA and structural pathologies.
2. Dilated funduscopic examination early in the evaluation of monocular visual loss is imperative to avoid unnecessary workup and diagnostic delays. Retinal and ocular imaging can also be very instructive in clarifying the diagnosis.
3. The 2022 American College of Rheumatology Classification Criteria for Giant Cell Arthritis can be a useful tool when evaluating a patient with signs and symptoms concerning for GCA; however, it is important to avoid premature application of the criteria to avoid anchoring bias.
4. The choroid is the most common ophthalmic structure affected by metastases due to its rich vascularity.

## AUTHOR CONTRIBUTIONS

**Laurel Ovrom:** Writing – original draft; writing – review and editing. **Zachary Pardieck:** Writing – original draft; writing – review and editing. **Randy Christopher Bowen:** Writing – original draft; writing – review and editing. **Neena R. Cherayil:** Writing – original draft; writing – review and editing; conceptualization.

## CONFLICT OF INTEREST STATEMENT

**Laurel Ovrom**, **Zachary Pardieck**, **Randy Christopher Bowen**, and **Neena R. Cherayil** declare no relevant conflicts of interest.
